# HIV integration sites in latently infected cell lines: evidence of ongoing replication

**DOI:** 10.1186/s12977-016-0325-2

**Published:** 2017-01-13

**Authors:** Jori Symons, Abha Chopra, Eva Malantinkova, Ward De Spiegelaere, Shay Leary, Don Cooper, Chike O. Abana, Ajantha Rhodes, Simin D. Rezaei, Linos Vandekerckhove, Simon Mallal, Sharon R. Lewin, Paul U. Cameron

**Affiliations:** 1 The Peter Doherty Institute for Infection and Immunity, University of Melbourne and Royal Melbourne Hospital, 792 Elizabeth St, Melbourne, VIC 3000 Australia; 2 Institute for Immunology and Infectious Diseases (IIID), Murdoch University, Murdoch, WA Australia; 3 HIV Translational Research Unit, Department of Internal Medicine, Faculty of Medicine and Health Sciences, Ghent University Hospital, Ghent University, Ghent, Belgium; 4 Department of Pathology, Microbiology and Immunology, Vanderbilt University, Nashville, TN 37232 USA; 5 Department of Infectious Diseases, Alfred Hospital and Monash University, Melbourne, Australia

## Abstract

**Background:**

Assessing the location and frequency of HIV integration sites in latently infected cells can potentially inform our understanding of how HIV persists during combination antiretroviral therapy. We developed a novel high throughput sequencing method to evaluate HIV integration sites in latently infected cell lines to determine whether there was virus replication or clonal expansion in these cell lines observed as multiple integration events at the same position.

**Results:**

We modified a previously reported method using random DNA shearing and PCR to allow for high throughput robotic processing to identify the site and frequency of HIV integration in latently infected cell lines. Latently infected cell lines infected with intact virus demonstrated multiple distinct HIV integration sites (28 different sites in U1, 110 in ACH-2 and 117 in J1.1 per 150,000 cells). In contrast, cell lines infected with replication-incompetent viruses (J-Lat cells) demonstrated single integration sites. Following in vitro passaging of the ACH-2 cell line, we observed a significant increase in the frequency of unique HIV integration sites and there were multiple mutations and large deletions in the proviral DNA. When the ACH-2 cell line was cultured with the integrase inhibitor raltegravir, there was a significant decrease in the number of unique HIV integration sites and a transient increase in the frequency of 2-LTR circles consistent with virus replication in these cells.

**Conclusion:**

Cell lines latently infected with intact HIV demonstrated multiple unique HIV integration sites indicating that these cell lines are not clonal and in the ACH-2 cell line there was evidence of low level virus replication. These findings have implications for the use of latently infected cell lines as models of HIV latency and for the use of these cells as standards.

**Electronic supplementary material:**

The online version of this article (doi:10.1186/s12977-016-0325-2) contains supplementary material, which is available to authorized users.

## Background

Despite the success of suppressive combination antiretroviral therapy (cART), HIV persists as integrated provirus in long lived latently infected cells, typically resting memory CD4^+^ T-cells [1, 2]. Latently infected memory CD4^+^ T-cells are rare in individuals on cART, occurring at a frequency of 10–100 per million cells [3], and therefore, are difficult to study ex vivo. Multiple in vitro models of HIV latency have been developed including latently infected cells lines and primary T-cells [4]. Understanding the location and frequency of HIV integration in the host genome in models of HIV latency as well as resting CD4^+^ T-cells from HIV-infected individuals on cART can potentially provide insights into the origin of infection, clonal expansion and potentially the response to latency reversing agents [5].

Latently infected cell lines are established following infection with either intact, replication-competent virus or mutated, replication-defective viruses. Examples of cell lines infected with replication competent virus include U1, ACH-2 and J1.1 cells [6–9] and with replication defective virus include J-Lat, where the cell lines are monoclonal and harbour a single integration site [10, 11]. In CD4^+^ T-cells from HIV-infected individuals on cART, several groups have recently shown a significant expansion of latently infected cells with a distinct site of integration, consistent with clonal expansion in vivo [5, 12–14]. Understanding whether similar patterns of integration occur in in vitro models of HIV latency and in patient derived cells is important, if these models are to be used to study the establishment, maintenance and reversal of latency.

Strategies to determine sites of HIV integration include sequencing and cloning [15, 16] or bulk sequencing [5, 12, 13, 17]. Most bulk sequencing approaches use restriction enzymes or random shearing of genomic DNA followed by PCR, using primers in the long terminal repeat (LTR) and a linker [5, 12, 13, 17]. Random shearing leads to different sized PCR products. Therefore, if an identical HIV integration site is detected but the length of the PCR product is different, it is most likely that this HIV integration sites was derived from a clonally expanded cell. Another method of determining the frequency of HIV integration sites is by limiting dilution of genomic DNA based on the estimated copies of HIV integrated DNA followed by loop amplification, and sequencing using primers located in the LTR [14].

Here, we describe a method to significantly streamline the assessment of HIV integration sites using robotic processing. Using this method, we evaluated HIV integration sites in commonly used latently infected cell lines and demonstrated that multiple cell lines that are traditionally used to study latency have evidence of productive infection.

## Methods

### Latently infected cell lines

Cells were obtained from NIH AIDS reagent program (Table 1) and were maintained in culture medium (CM) (RPMI 1640 medium (Life Technologies) supplemented with 10% (v/v) heat inactivated FCS, 100 μg/ml penicillin, 100 μg/ml streptomycin (Life Technologies) at 37 °C and 5% CO_2_. Cells were divided in a ratio of 1:6 or 1:10 twice weekly.

**Table 1:** Cell lines analysed in this study from NIH AIDS reagent program

Cell line	Replication competent virus	Site of mutation	Proviral copies reported per cell	Reference
U1	Yes	Mutation in tat	2	[6]
ACH-2	Yes	Mutation in tar	1	[7, 8]
J1.1	Yes	Wild type	1	[9]
J-Lat 8.4	No	Frame shift env	1	[10]
J-Lat 9.2	No	Frame shift env	1	[10]
J-Lat 10.6	No	Frame shift env	1	[10]
J-Lat 15.4	No	Frame shift env	1	[10]
J-Lat tat-GFP 8.2	No	LTR-tat-GFP	1	[10, 11]
J-Lat tat-GFP A1	No	LTR-tat-GFP	1	[10, 11]
J-Lat tat-GFP H2	No	LTR-tat-GFP	1	[10, 11]
J-Lat tat-GFP A72	No	LTR-tat-GFP	1	[10, 11]

*ENV* envelope, *LTR* long terminal repeat, *tat* trans-activator of transcription, *GFP* green fluorescent protein

### Sample collection for integration site analysis

To assess HIV integration sites in a panel of latently infected cell lines, all cells were passaged ten times in a 1:6 dilution twice weekly. High molecular weight genomic DNA (gDNA) was isolated from every other passage (i.e. passages 0, 2, 4, 6, 8 and 10) according to manufacturer’s protocol (Blood & Cell Culture DNA Mini Kit, QIAGEN). Passage zero was before the start of culture and sample was obtained from cryovials received from the NIH AIDS reagent program. To assess the efficiency of the HIV integration site analysis method, we spiked 1.5 µg high molecular weight gDNA from (peripheral blood mononuclear cells (PBMC) corresponding to approximately 2 × 10^5^ cells) derived from an HIV negative donor with high molecular weight gDNA from J-Lat 8.4, J-Lat 15.4 and J-Lat tat-GFP 82 cell line corresponding to a total of 125 cells. To test specificity, we analysed 1.5 µg gDNA PBMC from an HIV negative donor.

In some experiments ACH-2 cells were cultured in CM with and without 1 µM raltegravir (NIH AIDS reagent program). Cells were passaged nine times in a 1:10 dilution twice weekly and between passage 4 and 12, high molecular weight gDNA was isolated according to manufacturer’s protocol (Blood and Cell Culture DNA Mini Kit, QIAGEN). HIV integration sites and frequency of 2-LTR circles were then quantified.

### High throughput HIV integration analysis method

High molecular weight DNA corresponding to 1.5E10^5^ cells was randomly digested for 20 min at 22 °C with NEBNext dsDNA fragmentase according to manufacturer’s protocol (New England Biolabs, Ipswich, MA) to obtain short 200–1200 bp fragments for processing. Digested DNA was purified with AMPure XP (Agencourt Beckman Coulter, Nyon, Switzerland) in a 0.7:1 ratio, to remove small DNA fragments (<200 bp), buffer and fragmentase. The product was end-repaired, A-tailed and 10 pmol of linker (Additional file 1: Table S1) was ligated to the product using End-repair, ligation module (New England Biolabs) according to the manufacturer’s instructions. After purification, restriction digest was performed with BglII (New England Biolabs) to remove the upstream sequence of HIV and prevent sequencing of proviral HIV DNA. Purified product was amplified using a nested approach. The first round PCR was two staged with a first step of LTR dependent linear amplification with the biotinylated primer LTR1_F (Additional file 1: Table S1) for 98 °C for 3 min, followed by 12 cycles of 98 °C for 1 min, 60 °C for 30 s and 72 °C for 1 min, and then 10 min at 72 °C. In the second step, the PCR reaction was spiked with 1 µL 10 µM linker primer, 1stLink_Primer (Additional file 1: Table S1) that could only anneal when there was LTR dependent elongation for increased specificity, and then the same cycling conditions for 27 cycles. After purification of the PCR product with the AMPure XP (Agencourt Beckman Coulter) to remove unbound biotinylated primers, biotinylated PCR products were selected by streptavidin-coupled Dynabead selection according to the manufacturer’s protocol (Thermo Fisher, Malaga, Australia). Subsequently, a second round PCR was performed with the same cycling conditions for 35 cycles with the mid tagged primers LTR2_F and 2ndLink_Primer (Additional file 1: Table S1). Nested PCR was performed with Gotaq hotstart polymerase M5006 chemistry according to the manufacturer’s protocol (Promega, Madison, WI).

The purified products were quantitated using Nanodrop (Thermo Fisher, Malaga, Australia). Post quantification, up to 12 samples were pooled in equimolar amounts and a sequence library was prepared using the Kapa Hyper Library prep kit (Kapabiosystems, Wilmington, MS) using 500 ng of pooled product. The library was further quantified using qPCR and sequenced by 300 bp paired-end sequencing on Illumina MiSeq (Additional file 1: Fig. S1a) at appropriate concentrations. All the steps in the HIV integration site analysis methods were optimized for robotic processing to minimize contamination risk and increase throughput.

### Data analysis

To determine an HIV integration site, the Miseq reads were paired and needed to contain a multiplex-identifier (MID) sequence on either end of LTR and linker based sequence products. These sample read pairs were subsequently checked for LTR primer sequence and the remaining LTR nucleotide sequence ending on the nucleotide sequence CA. No mismatches were allowed in this sequence excluding CA (2 in primer sequence, 1 in LTR end). Furthermore, the linker primer had to be present. The sequence of the matched read pairs after the LTR or linker were trimmed to 50 base pairs and subsequently the trimmed read pairs were grouped according to 100% sequence match. Grouped sequences had to have ≥3 reads to proceed. Well represented sequences were used for chromosomal alignment which was determined using the Blat-UCSC Genome Browser (GRCH38/hg38). An HIV integration site was called if the results had ≥10 reads, and the frequency was determined by a length difference of ≥2 nucleotides (Additional file 1: Fig. S2).

### HIV DNA sequencing

DNA was extracted from pelleted ACH2 cells using QIAamp DNA Blood Mini kit (QIAGEN Inc., Hilden, Germany). Two overlapping amplicons spanning the HIV near full length genomes were PCR amplified using nested PCR with Platinum Taq DNA High Fidelity polymerase (Invitrogen, Carlsbad, CA). Each 25 μl reaction mixture contained 2.5 μl reaction mix (10×), 0.5 μl of each primer (0.5 mM), 0.25 μl Platinum TaqHigh Fidelity mix, 1.5 μl MgSO4 (3 mM), 1.25 μl dNTP mix (2 mM) and 2.5 μl of template DNA. The two step cycling conditions were 94 °C for 2 min; 15 cycles of 94 °C for 15 s, 60 °C for 20 s, and 68 °C for 6 min, 20 additional cycles of 94 °C for 15 s, 65 °C for 20 s, 68 °C for 6 min and finally 68 °C for 10 min. Amplicons were verified by agarose gel electrophoresis and quantified using Nanodrop. Amplicons were pooled in equimolar amounts. 500 ng of pooled product was used for preparing the indexed libraries for 454FLX and Illumina Miseq platforms. For 454FLX, the library was prepared using the GS FLX Titanium Rapid Library Preparation Kit as per manufacturer’s protocol (Roche 454 Life Sciences, Branford, CT). Library was quantified using the Kapa library quantification kit with 454FLX primer premix (Kapa Biosystems) and sequenced on the 454 FLX Titanium XL + instrument (454 Life Sciences. Roche Applied Sciences, Branford, CT). For Illumina sequencing, indexed libraries was prepared using the PCR free Kapa Hyper Prep kit as per the manufacturer’s protocol (Kapa Biosystems). The libraries were quantified using the KAPA Library Quantitation qPCR kit with Illumina Primer Premix (Kapa biosystems) and sequenced on Illumina MiSeq using 600 cycle MiSeq reagent kits (version 3). We analysed transition and transversion mutation additionally to insertions and deletion in the envelope gene with these two platforms. We called a mutation if the minority read count exceeded 1% of the total read count.

### 2-LTR circles and CCR5

2-LTR circles were measured as previously described [18]. Briefly; 2-LTR circle sequences were amplified by nested PCR with the first round PCR conditions including a denaturation step of 8 min at 95 °C and 16 cycles of amplification (95 °C for 30 s, 55 °C for 30 s, 72 °C for 1 min), followed by an elongation step of 5 min at 72 °C and the second round denaturation step of 10 min at 95 °C and 40 cycles of amplification (95 °C for 10 s, 60 °C for 30 s) followed by an elongation step of 5 min at 72 °C.

The number of 2-LTR circles were normalized to cellular input by preforming a separate real-time PCR assay to quantify the CCR5 coding sequence as previously described [19].

### Statistical analysis

The numbers of unique integration sites of all passages were scored independent of frequency. Differences during passaging were assessed using linear regression models. Differences in 2-LTR circles when the ACH-2 cell line was cultured with and without raltegravir was analyzed by a one-tailed paired *t* test. Differences in the decay of the number of ACH-2 integration sites with and without raltegravir were determined by regression analysis. Differences were considered statistically significant at *p* < 0.05. Gene ontology analysis was performed using the GEne SeT Analysis Toolkit [20]. Differences in ontology were analysed by Chi square test. All statistical analysis was performed using GraphPad Prism 6 and STATA.

## Results

### Development of a novel high throughput assay to study HIV integration

We assessed the efficiency of a novel high throughput method to study HIV integration using a mix of uninfected (Jurkat and PBMC from HIV-uninfected donors) and infected cell lines (J-Lat 8.4, J-Lat 15.4 and J-Lat tat-GFP 82). We detected 24–30 of 125 (19–24%) integration sites in a background of 1.5 µg gDNA from PBMCs from an HIV negative donor (n = 3). To test the specificity of our assay, we assessed HIV integration sites in 1.5 µg of gDNA derived from PBMCs from an HIV negative donor and no HIV integration sites were detected (data not shown, n = 3). We included several modifications to a recently published method [12] (Additional file 1: Fig. S1b), but the main adaptation that improved throughput was enzymatic random digestion with NEBNext dsDNA fragmentase, based on previous reports showing similar efficacy in DNA fragmentation to sonication [21]. After fragmentation, the use of nd-repair ligation module (New England Biolabs) which is a single step allowed ligation without product clean-up and therefore reduced the number of steps considerably. Furthermore, in our protocol we omitted linker ligation. By not digesting the linker, the second round nested PCR could also be performed with barcoded linker primers. Therefore the reverse reads were barcoded and sample specific. These changes enabled the whole procedure to be performed in a 96 well format allowing for up to 96 samples to be analysed in 16 h, which was run entirely on a robotic platform, making sample processing consistent. Only sample preparation was performed manually which meant that on average integration site analysis of 6-8 samples could be completed in 24 h.

### Diverse integration sites in latently infected cell lines infected with intact replication-competent viruses

**Fig. 1:**
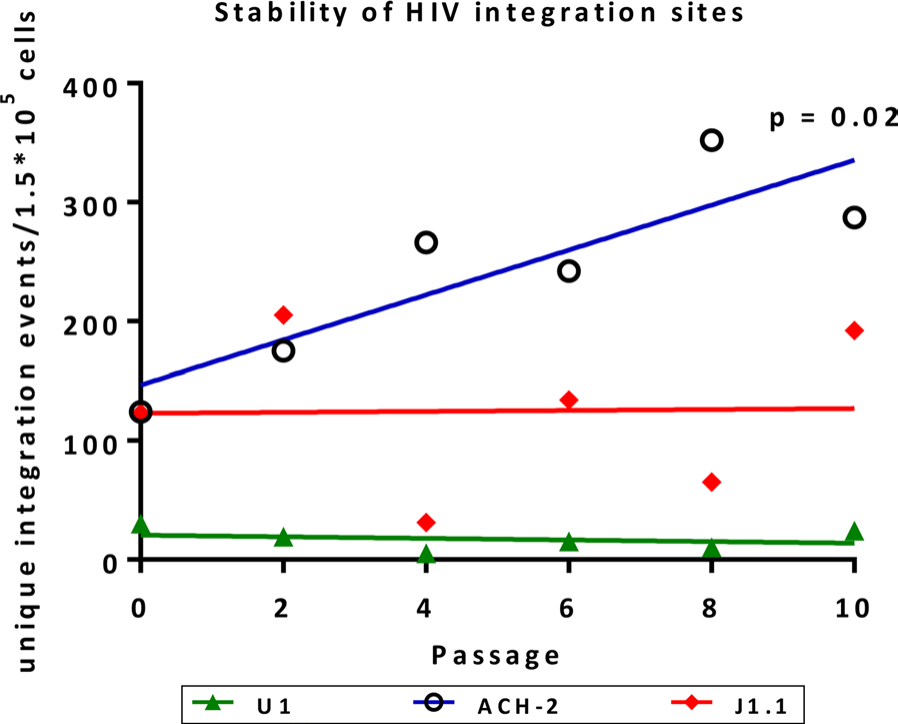
Change in frequency of HIV integration sites following passaging of cell lines infected with replication-competent HIV. The frequency of unique integration sites per 150,000 cells is shown following passaging of the U1 (*green*), ACH2 (*blue*) and J1.1 (*red*) cell lines. Linear regression was used to determine if the change was statistically significant

We next analysed HIV integration sites in established latently infected cell lines generated using intact replication-competent viruses or defective, replication-incompetent viruses. Cell lines reported to be infected with a replication-competent virus demonstrated multiple unique integration sites (number of unique integrations sites per 150,000 cells was 24–30, 89–124 and 123 in U1 (n = 2), ACH-2 (n = 2) and J1.1 cells (n = 1) respectively). Whereas the number of total integration sites detected in 150,000 cells was 667–754, 589–649 and 577 in U1 (n = 2), ACH-2 (n = 2) and J1.1 cells (n = 1) respectively). In contrast, all cell lines infected with a replication-incompetent virus (i.e. J-Lat cell lines) had a single unique integration site. This observation raised the possibility that there was ongoing infection of uninfected cells during cell passaging. In the ACH-2 cell line, the number of unique integration sites significantly increased over time in culture (*p* = 0.02), whereas it remained stable in J1.1 and U1 (Fig. 1). In contrast, all HIV integration sites in the J-Lat cell lines remained stable over time and were restricted to a single unique site (Table 2) (with a median of 388 (range 265–413) of total integration sites detected per 150,000 cells). The HIV integration sites in J-Lat cells 8.4, 9.2 and 15.4 were detected as previously reported [22].

**Table 2:** HIV integration sites in latently infected cell lines

Cell line	Replication competent virus	Chromosome^a^	Position^b^	Gene^c^	Gene orientation^d^	HIV orientation^e^	Percentage of events^f^
U1	Yes	2	48,177,527	AC079807.4	−	+	≈38%
		X	38,811,467			−	≈55%
		19	34,452,847	UBA2	+	+	≈3%
ACH-2	Yes	7	33,019,791	NT5C3A	−	−	≈37%
		9	128,111,651	SLC25A25-AS	−	−	≈13%
		2	26,386,668	EPT1	+	+	≈2%
		17	81,558,479	NPLOC4	+	−	≈1%
J1.1	Yes	11	685,243	DEAF1	−	−	≈41%
		12	54,257,973	CBX5	−	−	≈38%
J-Lat 8.4	No	1	7,946,384	FUBP1	−	−	100%
J-Lat 9.2	No	19	4,381,104	PPP5C	+	+	100%
J-Lat 10.6	No	9	136,468,579	SEC16A	−	+	100%
J-Lat 15.4	No	19	34,441,293	UBA2	+	+	100%
J-Lat tat-GFP 8.2	No	10	39,936,068			+	100%
J-Lat tat-GFP A1	No	X	34,073,326			−	100%
J-Lat tat-GFP H2	No	2	171,821,429	SLC25A12	−	+	100%
J-Lat tat-GFP A72	No	X	45,038,538	KDM6A	+	−	100%

^a^The particular chromosome where the HIV integration site is located

^b^The first human genome nucleotide following 3′-end LTR

^c^The gene where HIV is integrated

^d^Transcription direction of the gene

^e^Transcription direction of HIV

^f^Percentage of events where the particular HIV integration site was detected in all passages

**Fig. 2:**
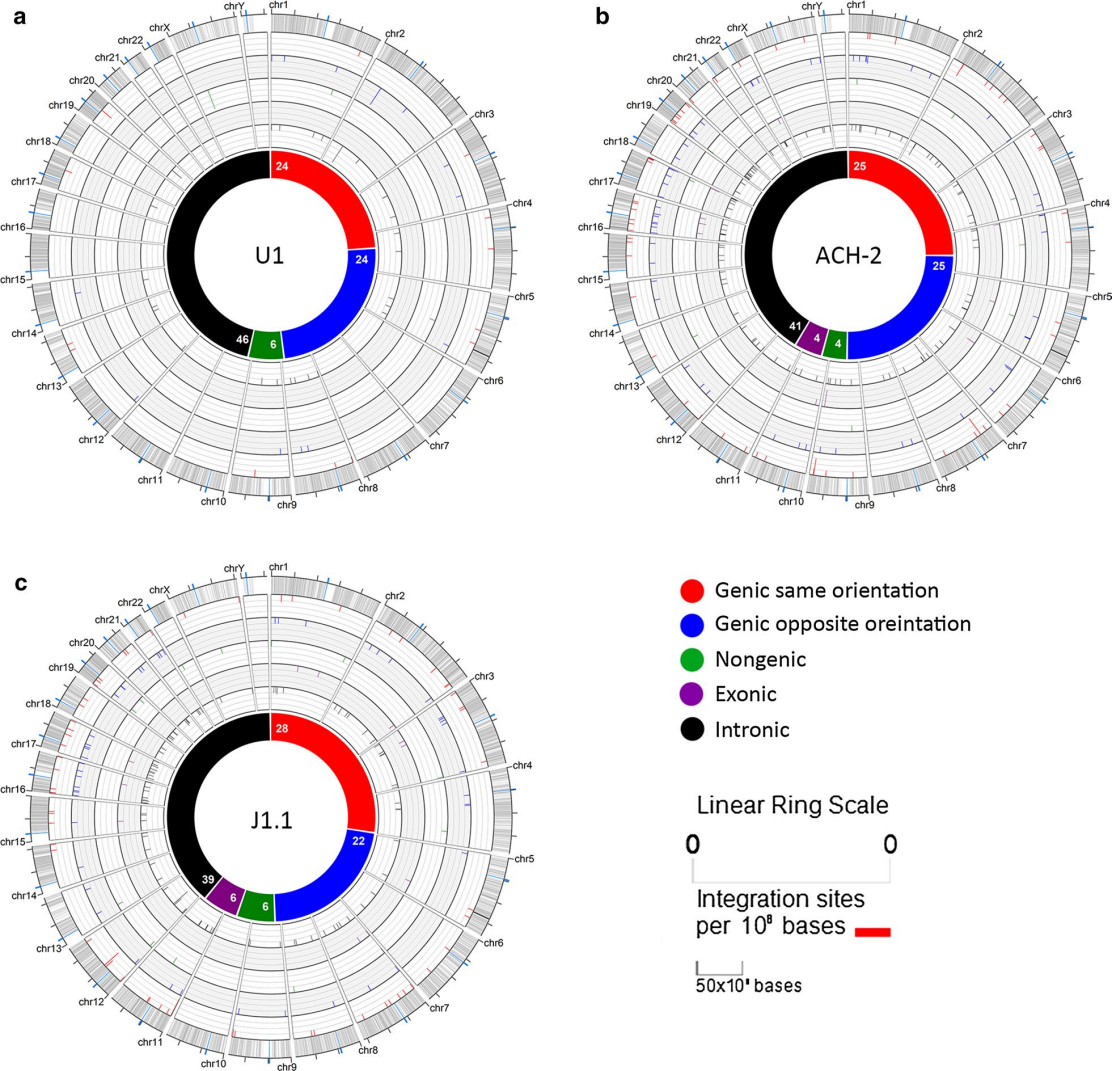
Site, frequency and nature of HIV integration sites in latently infected cells lines. A logarithmic depiction of HIV integration sites per million bases prior to passaging is shown for **a** U1, **b** ACH-2, and **c** J1.1. Chromosome numbers are labelled on the perimeter of the *outer circle*. The *outer circle* represents the different chromosomes with gene dense regions in *grey bars* representing different genes and in *blue* the centromere. The chromosomes are scaled to 50 million basepair bins. The integration sites for each chromosome are cumulative per million bases of the chromosome. The integration sites are shown as a coloured lines pointing to the centre of the circle. The frequency is represented in a logarithmic scale. The inner multi-colored *solid circle* summarises the nature of all the integration sites as genic (same or opposite orientation), non-genic, exonic and intronic. The specific site of integration is shown for each chromosome with the first ring showing integration within a gene with the same orientation compared to the host gene [genic (same orientation)] in *red*. The second ring shows HIV integration sites and within a gene with the opposite orientation compared to the host gene [genic (opposite orientation)] in *blue*. The *third ring* shows HIV integration sites not in a gene (non-genic) in *green*. In the fourth ring HIV integration sites in an exon (exonic) are in *purple*. In the fifth ring HIV integration sites in an intron (intronic) are in *black*

We also analysed the orientation of integrated HIV and if there were differences in integration within genes (intron, exon) or in nongenic regions in the U1, ACH-2 and J1.1 cell lines from passage zero and passage ten. We found that most integration sites were within genes and specifically introns, and there was no preference in orientation of HIV integration and this did not change following passaging (Fig. 2).

### Identity of genes where HIV integrates in latently infected cell lines

We next analysed the specific gene locations of HIV integration sites that were frequently detected during in vitro passaging. In the U1 cell line, we detected two major sites with high frequency. One site was in chromosome 2 in the *AC079807.4* gene with the 3′ LTR junction at nucleotide position 48,177,527 whereas the other site was found in chromosome X in a functionally unknown region. Interestingly, an additional site was continuously detected in chromosome 19, in the ubiquitin-like modifier activating enzyme 2 (*UBA2*) gene, in about 4 percent of events that were detected (Table 2).

In the ACH-2 cell line, the major HIV integration site was detected in chromosome 7 in the cytosolic 5′-nucleotidase 3A (*NT5C3A*) gene as previously reported [17, 23]. We also detected an additional integration site in chromosome 9 which was always detected in high frequency in the solute carrier family 25 antisense RNA (*SLC25A25*-*AS*) noncoding RNA gene as recently reported [17]. Additionally, we detected two sites with multiple integration events intermittently, one in chromosome 2 in the ethanolaminephosphotransferase 1 (*EPT1*) gene, the other in chromosome 17 in the nuclear protein localization 4 (*NPLOC4*) gene (Table 2).

Although previous reports have described a single copy of integrated HIV per cell in the J1.1 cells [9], we detected two major integration sites in 150,000 cells with equal frequency in passage zero before start of cell culture. One was detected in chromosome 11 in the deformed epidermal autoregulatory factor 1 (*DEAF1*) gene and the other in chromosome 12 in the chromobox homolog 5 (*CBX5*) gene (Table 2). During the ten passages, the HIV integration site in all the J-Lat cell lines remained single and stable and the identified integration sites represented 100% of the total events detected (Table 2).

**Fig. 3:**
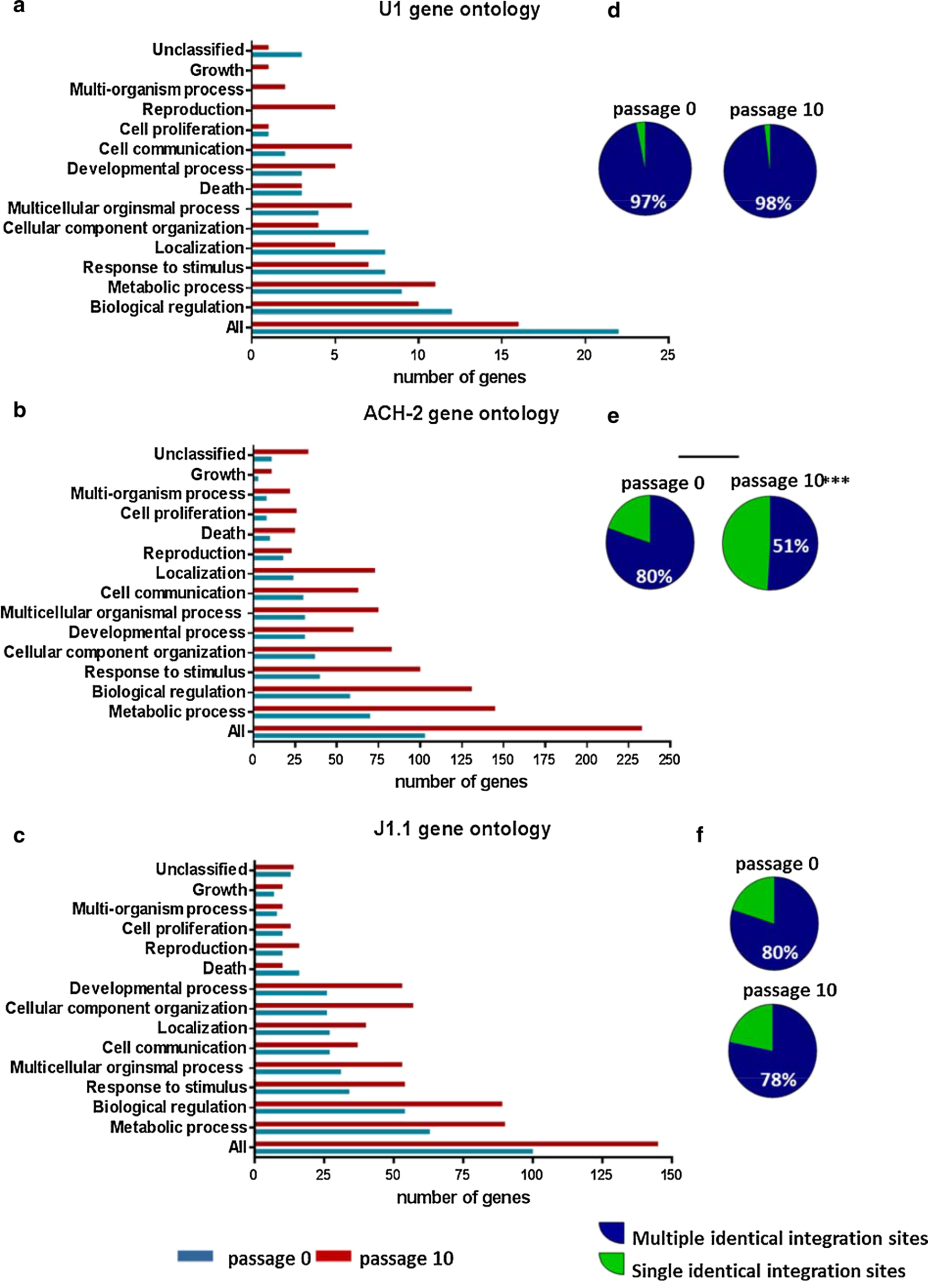
Gene ontology analysis of latently infected cell lines. The gene ontology of HIV integration sites detected in the **a** U1, **b** ACH-2 and **c** J1.1 cell lines using Gene SeT Analysis Toolkit is shown as the number of genes in a distinct family for passage 0 (*blue bar*) and passage 10 (*red bar*). The proportion of HIV integration sites detected more than once (*blue*, multiple integration events) or on one occasion (*green*, single integration event) in the **d** U1, **e** ACH-2 (3e) and **f** J1.1 cell lines at passage 0 (p0) and 10 (p10) is shown as a pie chart

We analysed the genes where HIV integrated following passage zero and passage ten for the U1, ACH-2 and J1.1 cell lines. We observed that with passaging, the number of HIV integration sites in genes decreased in the U1 cell line (Fig. 3a), but increased in ACH-2 (Fig. 3b) and J1.1 (Fig. 3c) cell lines. The proportion of integration sites in genes associated with cellular proliferation was approximately 10% and did not significantly differ between the passages in all cells.

We then compared the integration sites in the cell lines with those found in CD4^+^ T-cells from HIV-infected individuals on cART, which have been previously reported in the literature [5, 12–14, 24]. The number of integration sites we detected in the U1, ACH-2 and J1.1 cell lines overlapped 22, 33 and 35% respectively when compared to HIV integration sites found in cells from HIV-infected individuals on ART. This suggests that many sites of HIV integration identified in latently infected cell lines were not identified in cells from HIV-infected individuals on ART.

### Multiple identical integration sites in latently infected cell lines

We analysed the percentage of identical integration events compared to total integration sites per cell line. Identical integration sites were defined as two or more integration events. In the U1 cell line, which contains an intact virus, most integration sites were identical and detected multiple times (97% of all integration sites detected were identical at passage 0) and did not change during passage (98% at passage 10) (Fig. 3d). In the ACH-2 cell line, we observed a significant decrease in the percentage identical integration events (from 80% at passage 0–51% at passage 10, *p* < 0.001) (Fig. 3e). In the J1.1 cell line, the percentage of identical integration events did not significantly differ during passage (80% at passage 0, 78% at passage 10) (Fig. 3f).

### Change in integration sites over time in culture is consistent with residual virus replication in latently infected ACH-2 cell line

To determine if the observed effects in the ACH-2 cell line were due to ongoing virus replication, we sequenced the integrated provirus from the ACH-2 cell line from passage 1 with two deep sequencing methods and analysed the envelope gene and demonstrated multiple transition and transversion mutations, insertions and deletions with 454 sequencing. Moreover with illumina Miseq sequencing we observed multiple transition (50) and transversion [36] mutations, insertions [8] and deletions [5] With 70–80% of the mutations,insertions and deletions detected in the Miseq data overlapping with the mutations found by 454 sequencing.

**Fig. 4:**
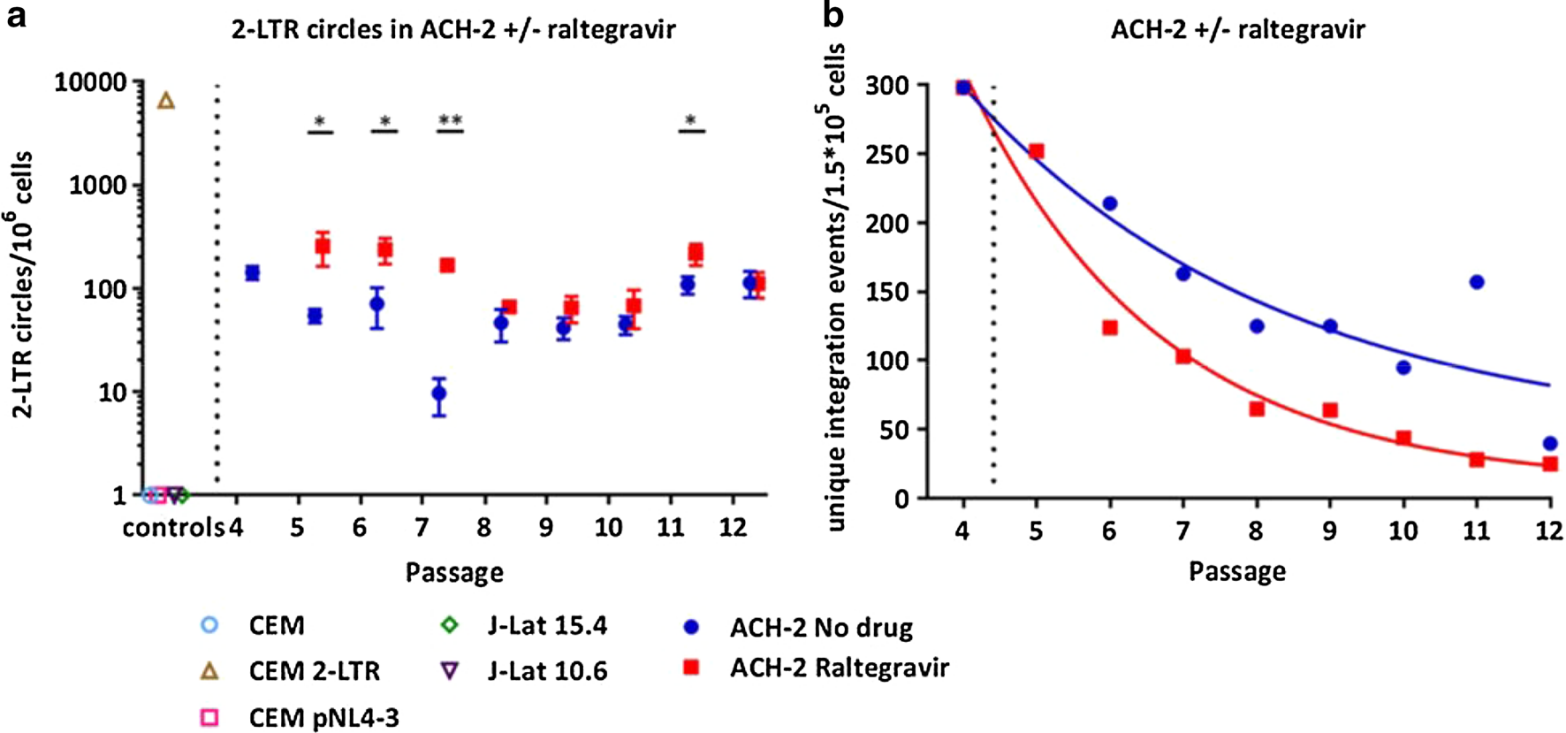
The effect of raltegravir on integration sites and 2-LTR circles using the ACH-2 cell line. The ACH2 cell line was passaged multiple times in the presence (*red*) and absence (*blue*) of raltegravir and **a** the frequency of 2-LTR circles per million cells and **b** number of unique integration sites were quantified. Positive controls included CEM cells spiked with 2-LTR circles; negative controls were CEM cells, CEM cells spiked with pNL4-3, J-Lat 10.6 and J-Lat15.4. *p = 0.018 and 0.02, **p = 0.004

Subsequently, we analysed the frequency of 2-LTR circles in the ACH-2 cell line cultured with and without 1 µM raltegravir. Surprisingly, in the ACH-2 cell line, we detected 2-LTR circles in cells collected from each passage. We detected significantly more 2-LTR circles per million cells in the first three passages with raltegravir compared to without raltegravir (*p* = 0.04, *p* = 0.02 and *p* < 0.01 for the first three passages compared to baseline). As expected, 2-LTR circles were detected in the positive control of the CEM cell line spiked with the 2-LTR plasmid while 2-LTR circles were not detected in the uninfected CEM cell line, in the CEM cell line spiked with pNL4-3 or the J-Lat 15.4 and 10.6 cell lines (Fig. 4a). We observed a decrease in the number of unique integration sites over time following twice weekly passage and a faster decay in the number of HIV integration sites in the ACH-2 cell line cultured with raltegravir compared to without raltegravir (half-life of number of integration sites per passage was 1.81 versus 2.96 passage respectively) although the difference was not statistically significant (*p* = 0.57) (Fig. 4b). Moreover, we observed a consolidation around the reported dominant integration site in chromosome seven from 32% in passage four to 74% in passage 12.

## Discussion

In this study, we developed a high-throughput assay to analyse HIV integration sites using enzymatic random fragmentation. Our assay was specific and was able to detect 25% of the HIV integration sites using infected cell lines (J-Lats) in a background of human gDNA. In cell lines infected with replication-incompetent viruses, we observed single integration sites. In contrast, in cell lines infected with replication-competent viruses, we demonstrated multiple unique HIV integration sites and in the ACH-2 cell line, the diversity of integration sites increased over time in culture and reduced in the presence of the antiretroviral drug raltegravir. Therefore, these latently infected cell lines were not clonal and the findings were consistent with residual virus replication.

The U1 monocytic cell line was cloned from a population of chronically HIV-1 infected U937 cells and is one of the most-studied cell lines for HIV latency [7]. It has been reported that U1 cells contain two copies of integrated HIV DNA [7] and latency is maintained by mutations in Tat with one Tat containing a point mutation, H13L, and the other lacking the ATG initiation codon [25]. Our analysis demonstrated that the U1 cell line contains multiple distinct HIV integration sites that declined with passaging.

J1.1 cells are latently HIV infected cell line cloned by limiting dilution from HIV-infected Jurkat cells. Although, J1.1 cells are infected with wild type HIV_LAV_, the HIV-infected T-cell clone is defective in IL-2 production and Ca2^+^ mobilization after CD3 stimulation [9]. Furthermore, J1.1 cells are reported to be infected with a single integrated copy per cell [9]. Interestingly, we detected multiple HIV integration sites and identified two major integration sites in equal frequency. Why there is preferential expansion or persistence of these specific integration sites is unclear.

The ACH-2 cell line was established by infection of the acute lymphoblastic leukemic T-cell line A3.01 with HIV_LAI_. Cells that survived were cloned resulting in a cell line that has been reported to contain a single integrated copy of HIV [6, 8]. The major HIV insertion site has been identified in chromosome 7 [23]. In addition, HIV latency is established by a point mutation in TAR (C37T), whereby this mutation impairs the response of the LTR to Tat [26]. Low level transcription of multiply spliced transcripts persist in ACH-2 cells in the absence of stimulation [27], and here, we show that ACH-2 cells harbour multiple integration sites in addition to the major site in chromosome 7, as recently demonstrated by others [17].

Here, we present multiple lines of evidence consistent with residual virus replication in, ACH-2 cell line, and evidence suggestive for multiple rounds of replication occurring in the U1 and J1.1 cells. The evidence included diversity of integration sites in all three cell lines that was stable or increasing study directly links several lines of evidence demonstrating residual viral over time, and in the ACH-2 cell line, an increase in diversity during passage, numerous mutations in the proviral DNA and an increase in 2-LTR circles in the presence of raltegravir. Other groups have demonstrated viral production in the latently infected cell lines, U1, ACH-2 and J1.1 cells demonstrating continuous expression of viral RNA [28] and detection of 2-LTR circles in the ACH-2 cell line [29]. Our results are also consistent with the observed increase in integration sites in the HIV infected cell line H61-D during culture [30]. Olivares et al. demonstrated that during prolonged culture of these cells up to 100 passages, integration sites were detected in addition to the two sites found at passage zero [30].

Recently, Sunshine et al. [17] hypothesised that the presence of infrequent integration sites in the ACH-2 cell line indicated low-level viral replication. Our study presents several lines of evidence demonstrating residual viral replication in the latently infected cell line ACH-2. Additionally, our study is the first to suggest ongoing low level replication in othercell lines infected with replication-competent viruses commonly used to study HIV latency. It is important to note that there is no perfect model to study HIV latency in vitro. Latently infected cell lines remain valuable in the assessment of latency or in activation studies using latency reversing agents. The advantages of latently infected cell lines include a high frequency of infected cells, the capacity to repeat experiments reproducibly and the ability to answer specific mechanistic questions that often isn’t possible in primary cells.

It is possible that in our study, we increased the chance of cell-to-cell transmission of HIV by passaging cells at low dilution, which would allow for cell clumping. It has been previously demonstrated that cell-to-cell transmission increases with higher cell density [31, 32]. The degree of cell clumping could therefore have an impact on the level of residual virus replication in this cell line. Interestingly, the ACH-2 cell line is reported to not express CD4 on the cell surface, although transcription of CD4 can be induced [33]. Similarly, the H61-D cell line that demonstrated an increase in integration sites during culture did not express CD4 at the cell surface [30]. Cell-to-cell transmission can occur through a non-CD4 dependent process [34, 35] or CD4 could be expressed at low levels in the virological synapse.

Similar to other studies, we did not identify a high frequency of the same genes harbouring HIV integration in the latently infected cell lines that have been described in CD4^+^ T-cells from HIV-infected individuals on cART [5, 12–14, 24]. HIV integration in BACH2 and MKL2 [5, 14], although commonly found in patient derived cells were not detected in all the different cell lines. It has been suggested that the selective pressure for expansion of HIV integration sites into genes associated with cell proliferation is absent, since these cell lines have been previously immortalized. However, the proportion of HIV integration sites in genes associated with cell proliferation was similar in the U1, ACH-2 and J1.1 cell lines and in the CD4^+^ T-cells from HIV-infected individuals on cART [5, 14]. Similar findings were recently demonstrated also using the ACH-2 cell line as well as a primary T-cell latency model [17]. Given that the same orientation of HIV integration relative to the host gene increases transcriptional efficiency and latency [36], this might potentially explain the higher observed basal HIV reverse transcriptase activity in ACH-2 compared to the U1 cell line [37]. There were several limitations in our study. First, we only report HIV integration sites in latently infected cell lines. However, others have compared cell lines to primary cell models, and observed similar patterns of HIV integration with respect to increased integration in intronic regions and no specificity for HIV integration orientation [15, 38, 39]. In addition, there was some overlap in the sites of integration in the cell lines and published work on cells from HIV-infected individuals on cART [5, 12, 13, 24]. Second, we were not able to definitely demonstrate if these cells harboured multiple HIV integration sites per cell or whether these cell lines contained multiple cells with different HIV integration sites. We suspect that multiple integration sites per cell, in cell lines and in vivo is rare but can occur [30, 40–42]. Moreover, it seems unlikely that a new integration event in a cell line is associated with the loss of the dominant integration site.

## Conclusions

We present a specific and efficient new method to analyse the location and frequency of HIV integration sites suitable for high throughput processing. Using HIV latently infected cell lines we demonstrated that latently infected cell lines that are infected with a replication-competent HIV had multiple HIV integration sites, and that this was likely due to low level replication. These findings have implications for the use of some latently infected cell lines as models for HIV latency whereas they are not truly latent and one must take into account that some cells might have a different number of integrated proviruses per cell than previously thought when one uses these cells for standardization of assays.

## Additional file

**Additional file 1.** Schematic overview of methods used for HIV integration site analysis.
