# Erratum to: HIV integration sites in latently infected cell lines: evidence of ongoing replication

**DOI:** 10.1186/s12977-017-0340-y

**Published:** 2017-03-27

**Authors:** Jori Symons, Abha Chopra, Eva Malatinkova, Ward De Spiegelaere, Shay Leary, Don Cooper, Chike O. Abana, Ajantha Rhodes, Simin D. Rezaei, Linos Vandekerckhove, Simon Mallal, Sharon R. Lewin, Paul U. Cameron

**Affiliations:** 1 The Peter Doherty Institute for Infection and Immunity, University of Melbourne and Royal Melbourne Hospital, 792 Elizabeth St, Melbourne, VIC 3000 Australia; 2 Institute for Immunology and Infectious Diseases (IIID), Murdoch University, Murdoch, WA Australia; 3 HIV Translational Research Unit, Department of Internal Medicine, Faculty of Medicine and Health Sciences, Ghent University Hospital, Ghent University, Ghent, Belgium; 4 Department of Pathology, Microbiology and Immunology, Vanderbilt University, Nashville, TN 37232 USA; 5 Department of Infectious Diseases, Alfred Hospital and Monash University, Melbourne, Australia

## Erratum to: Retrovirology (2017) 14:2 DOI 10.1186/s12977-016-0325-2

In the original publication [1] the spelling of one author name was incorrect. The correct version:

Eva Malatinkova
