# Supplementary material for: HIV integration sites in latently infected cell lines: evidence of ongoing replication
Source: Retrovirology. 2017 Jan 13;14:2. doi: 10.1186/s12977-016-0325-2 (PMC5237276; doi:10.1186/s12977-016-0325-2)
Supplement: Supplementary file 1 — Additional file 1. Schematic overview of methods used for HIV integration site analysis. [file 12977_2016_325_MOESM1_ESM.docx]

**
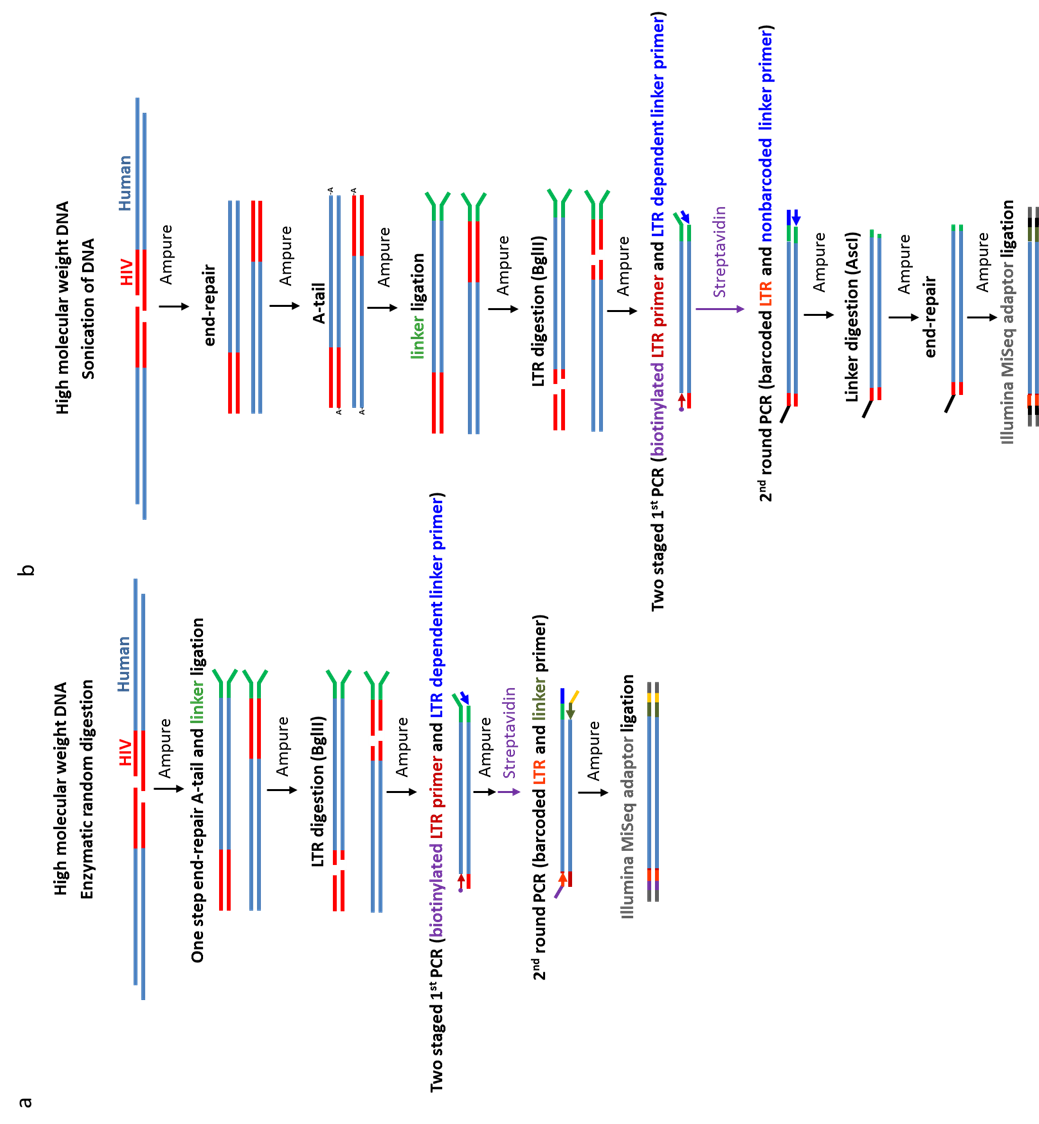
**

**Figure S1. Analysis of HIV integration sites.** Comparison of A. a new high throughput method suitable for robotic processing and B a published method [12] to analyse HIV integration sites.


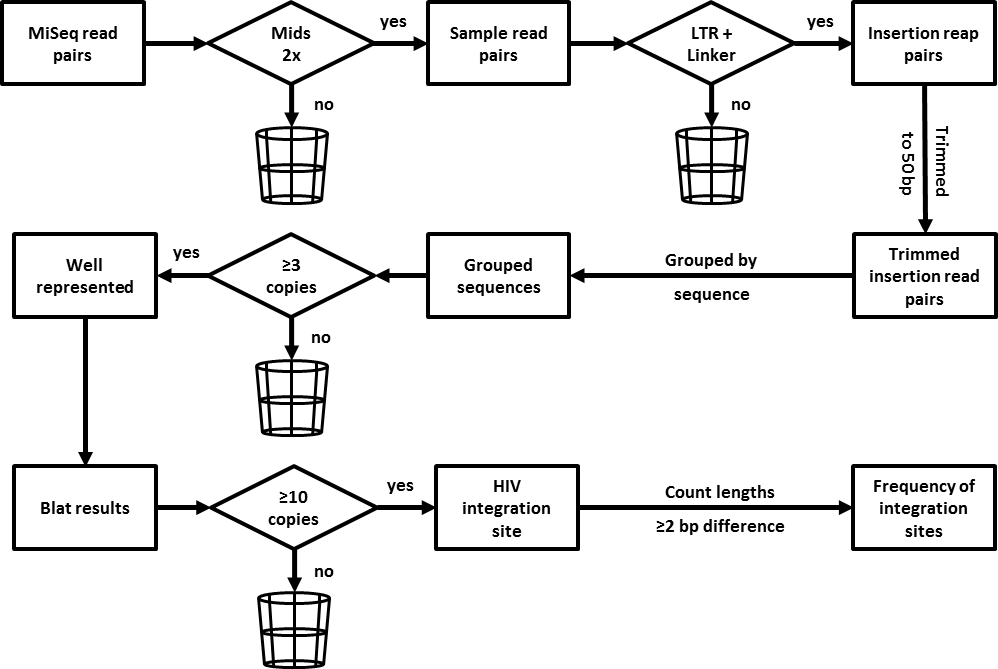


**Figure S2.** Schematic overview of analysis strategy used to define an integration site.

**Table S1.** Linker sequence and primer sequence.

* indicates phosphorothioated nucleotide. MID is multiplex identifier sequence.
